# Haptic Adaptive Feedback to Promote Motor Learning With a Robotic Ankle Exoskeleton Integrated With a Video Game

**DOI:** 10.3389/fbioe.2020.00113

**Published:** 2020-02-21

**Authors:** Guillermo Asín-Prieto, Aitor Martínez-Expósito, Filipe O. Barroso, Eloy J. Urendes, Jose Gonzalez-Vargas, Fady S. Alnajjar, Carlos González-Alted, Shingo Shimoda, Jose L. Pons, Juan C. Moreno

**Affiliations:** ^1^Neural Rehabilitation Group, Cajal Institute, CSIC–Spanish National Research Council, Madrid, Spain; ^2^Department of Information Systems Engineering, University San Pablo CEU, Boadilla del Monte, Spain; ^3^Department of Translations Research and Knowledge Management, OttoBock Healthcare GmbH, Duderstadt, Germany; ^4^College of Information Technology, The United Arab Emirates University, Al-Ain, United Arab Emirates; ^5^Centro de Referencia Estatal de Atención al Daño Cerebral, Madrid, Spain; ^6^Intelligent Behaviour Control Unit, RIKEN, Nagoya, Japan; ^7^Legs & Walking AbilityLab, Shirley Ryan AbilityLab, Chicago, IL, United States; ^8^Department of Biomedical Engineering and Mechanical Engineering, McCormick School of Engineering, Northwestern University, Chicago, IL, United States; ^9^Department of PM&R, Feinberg School of Medicine, Northwestern University, Chicago, IL, United States

**Keywords:** bioinspired, exoskeleton, video game, motor learning, corticospinal, plasticity, stroke, TMS

## Abstract

**Background:** Robotic devices have been used to rehabilitate walking function after stroke. Although results suggest that post-stroke patients benefit from this non-conventional therapy, there is no agreement on the optimal robot-assisted approaches to promote neurorecovery. Here we present a new robotic therapy protocol using a grounded exoskeleton perturbing the ankle joint based on tacit learning control.

**Method:** Ten healthy individuals and a post-stroke patient participated in the study and were enrolled in a pilot intervention protocol that involved performance of ankle movements following different trajectories via video game visual feedback. The system autonomously modulated task difficulty according to the performance to increase the challenge. We hypothesized that motor learning throughout training sessions would lead to increased corticospinal excitability of dorsi-plantarflexor muscles. Transcranial Magnetic Stimulation was used to assess the effects on corticospinal excitability.

**Results:** Improvements have been observed on task performance and motor outcomes in both healthy individuals and post-stroke patient case study. Tibialis Anterior corticospinal excitability increased significantly after the training; however no significant changes were observed on Soleus corticospinal excitability. Clinical scales showed functional improvements in the stroke patient.

**Discussion and Significance:** Our findings both in neurophysiological and performance assessment suggest improved motor learning. Some limitations of the study include treatment duration and intensity, as well as the non-significant changes in corticospinal excitability obtained for Soleus. Nonetheless, results suggest that this robotic training framework is a potentially interesting approach that can be explored for gait rehabilitation in post-stroke patients.

## 1. Introduction

Stroke affects each year around 13.7 million people worldwide, is the second leading cause of disability and may result in a series of motor impairments including gait abnormalities (Barroso et al., 2017; World Stroke Organization, 2018). Regarding walking rehabilitation after stroke, there has been considerable controversy and debate on the effectiveness of the various approaches used (Pollock et al., 2014). In the past 20 years, other rehabilitation modalities, such as robotic therapy have been introduced to motor rehabilitation practice aiming at promoting gait recovery in patients who suffered neural-impairments (Moreno et al., 2013), including post-stroke patients. So far, results suggest that robotic therapy may be beneficial to treat acute and chronic post-stroke patients (Van der Loos et al., 2016). Nonetheless, there is no agreement on the optimal robot-assisted approaches to promote neurorecovery through plasticity mechanisms following neural injury (Kim and You, 2017; Belas dos Santos et al., 2018; Gassert, 2018; Barroso et al., 2019).

One of the most widely tested approaches is robotic guidance, which supervises trajectories during motor tasks and prevents the user from performing undesired (and possibly unsafe) deviations from prescribed trajectories. This type of robotic assistance is frequently implemented as a “tunnel” of allowed deviation around the prescribed trajectory (Ren et al., 2011; Bortole et al., 2015). Robotic guidance can be combined with virtual environments or video games. Adding video games to the therapy turns the potential motor learning into a transparent process to the user. Moreover, engagement with the training and entertainment are very important psychological aspects of games (Patton and Mussa-Ivaldi, 2004). In fact, visual feedback has been shown to improve robotic guidance therapy scenarios (Liu et al., 2006; Tamburella et al., 2019) and video games seem to be effective to improve motor function and health after stroke (Swanson and Whittinghill, 2015). Thus, different combinations of robotic guidance and video games have been proposed. A possible shortcoming of robotic guidance is that this approach might as well reduce patients' effort, and thus, the possible benefits of the therapy (Rowe et al., 2017). In this vein, Goodman et al. (2014) designed a video game that decreased the level of assistance delivered to the ankle joint by the robot if the performance (assessed as a function of the smoothness of trajectories) increased. Other strategies involve adding resistance to make the task more challenging when the performance of the user improves, which can potentially increase engagement in the task (Ren et al., 2011). Interestingly, these two opposite strategies found evidence of enhanced motor learning markers, although there is still no consensus regarding the effects of using either type of robotic guidance.

As a counterpart of robotic guidance, error-augmentation based approaches have been also proposed to enhance motor learning. Emken and Reinkensmeyer (2005) used movement-perturbation approach with a robotic device while the user was performing the target task and concluded that motor learning can be accelerated by exploiting the error-based learning mechanism. Reinkensmeyer and Patton (2009) suggested that starting with guidance force and gradually removing it and increasing error-augmentation approaches may lead to motor learning. Marchal-Crespo et al. (2014) showed that adding random disturbances while executing a simple dorsi-plantarflexion task improved motor learning and suggested that the variability introduced to the task may increase recovery due to increased effort and attention needed to perform the task. Moreover, another study showed that “challenge-based” controllers (where guidance force is given on the first stages of the recovery and error-augmentation is given later on the rehabilitation) were more beneficial for the recovery, since this represents an adaptation of the therapy to the patients' motor learning process (Marchal-Crespo et al., 2017). These functional benefits observed in these studies suggest that the nervous system learns by forming the internal model of the dynamics of the environment via error reduction (Emken and Reinkensmeyer, 2005), leading to plastic changes presumably at the cortical level (Perez et al., 2004).

Given the aforementioned literature on different approaches tested in robotic therapy, there is evidence supporting the integration of video games in challenge-based therapies, that are able to adapt the difficulty of the task to the patient's skills, always trying to keep the user motivated and engaged. This might help promoting motor learning via activity-dependent neuroplasticity (Sweatt, 2016; Gassert, 2018). In this context, the present study proposes a novel therapy protocol that combines a grounded exoskeleton perturbing the ankle joint motion with a video game based visual feedback. Ankle joint is fundamental for gait and balance as plantarflexor passive stiffness causes reduced plantarflexion torque before starting the swing phase in gait, and may as well limit dorsiflexion, compromising foot clearance in post-stroke patients (Lamontagne et al., 2002). The major novelty that this therapy protocol introduces is the autonomous modulation of the perturbations provided to the user via haptic adaptive feedback approach based on the task performance. This protocol was first tested on a validation study with healthy subjects, and later on as an usability case study with a post-stroke patient.

We hypothesized that the use of the proposed ankle rehabilitation robot would promote motor learning and increase corticospinal excitability of the dorsi-plantarflexor muscles. Although there is not a clear relationship between motor learning and corticospinal excitability (Bestmann and Krakauer, 2015), several authors have established a relation between them (Perez et al., 2004; Kida et al., 2016; Naros et al., 2016; Mawase et al., 2017; Christiansen et al., 2018; Raffin and Siebner, 2018; Mrachacz-Kersting et al., 2019). Corticospinal excitability can be assessed with Transcranial Magnetic Stimulation (TMS), by eliciting Motor Evoked Potentials (MEPs) (Rotenberg et al., 2014). Validation of our hypotheses would provide preliminary evidence of the usefulness of this novel robotic therapy to promote motor learning in the context of a pre-gait mobilization task, i.e., mobilization before undergoing gait-centered rehabilitation.

## 2. Materials and Methods

### 2.1. Participants

Ten healthy subjects (29.80 ± 6.32 years old) participated in the study. They signed an informed consent for the experiment. Experiments were conducted in accordance with the declaration of Helsinki. All experimental procedures were approved by the Bioethical subcommittee of the Ethical committee of CSIC (Spanish National Research Council), reference 008/2016.

We also performed an usability case study with one post-stroke patient (age 37). The patient suffered an haemorrhagic transformation of ischemic stroke, affecting the right middle cerebral artery, thus the most affected side of the body was the left. The experiment with the patient was performed in the facilities, and under the supervision of the professionals of *Centro de Referencia Estatal de Atención Al Daño Cerebral (CEADAC)*. The patient was assessed by a physician using the most common scales: the Disability Rating Scale (DRS), Functional Independence Measure (FIM) and the Barthel Index (BI). For the DRS, the value was 2, corresponding with a partial level of disability. The BI score was 100, reflecting independence in the activities of daily living, while for the FIM it was 119 (85 for motor subscale and 34 for cognitive scale). The patient signed the Informed Consent, acknowledging the risks and the inclusion criteria (he was previously examined by a physician, who validated the suitability for the training). These experimental procedures were approved by the local scientific committee in *CEADAC*.

### 2.2. Experimental Platform

The Biomot ankle robot (Moltedo et al., 2016) was used in this study. Footedness preference for each subject was established according to the Waterloo footedness test (Elias et al., 1998).

**Figure 1:**
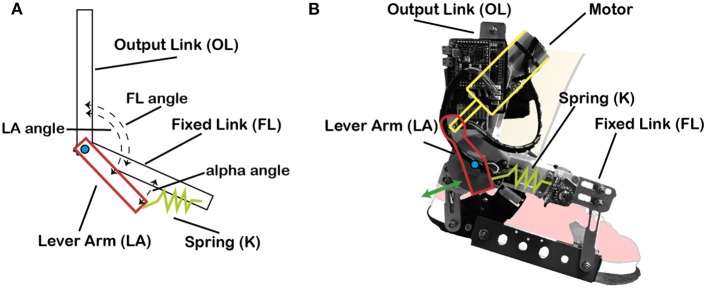
MACCEPA actuator model schematics and actual actuator. **(A)** MACCEPA actuator schematics, with all its components. **(B)** MACCEPA attached to a cartoon foot, with the different components depicted.

This actuator is based on the MACCEPA (mechanically adjustable compliance and controllable equilibrium position actuator) concept (Bacek et al., 2015), which is driven by a joint torque control. MACCEPA concept is based on a torque-controlled rotational actuator with adjustable compliance (Figure 1). The motor is rigidly connected to the Lever Arm (LA), which is in turn connected to the Fixed Link (FL) via a spring (K). FL is attached to the wearer's foot and thus its angle represents the user's ankle angular position, and LA represents the robot position. Both LA and FL move with respect to the Output Link (OL), which is attached to the wearer's shank. Consequently, if the motor reference is set to a particular position, the wearer still has the possibility to pivot the ankle by compressing the spring. This permits to calculate the interaction torque between the wearer and the actuator by measuring the subsequent deflection of the spring (α angle = *LA* − *FL*).

MACCEPA actuator allows to provide controlled torque profiles by using a simple position controller without the need of a complex torque sensor, and with the reliability of position sensors.

### 2.3. Robot Control

The controller of this robotic platform comprises a zero torque controller (based on a classic Proportional/Integral/Derivative (PID) implementation) and the haptic adaptive feedback (HAF) component based on tacit adaptability—a symbiotic control strategy on exoskeletons inspired by biomimetic mechanisms, which, in turn, is based on the “tacit learning” approach for bipeds (Shimoda et al., 2015; Asín-Prieto, 2016), adapted by the performance of the user in the experimental task.

**Figure 2:**
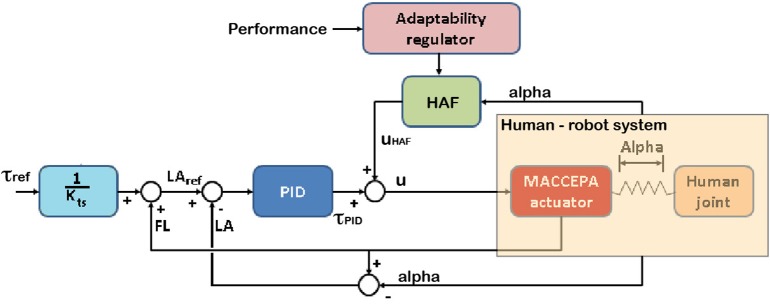
The controller of the robot comprises a zero torque Proportional/Integral/Derivative (PID) controller and the haptic adaptive feedback module (HAF constant—*K*_*HAF*_—multiplied by alpha, angle proportional to the interaction between the robot and the subject), tweaking *K*_*HAF*_ with the performance. The subject controls the location of the character on the screen by means of the ankle joint angle. In the figure, *u* stands for the output of the controller, τ_*ref*_ is the disturbance torque reference, τ_*PID*_ and *u*_*HAF*_ are respectively the outputs from the PID torque and HAF controllers; and *FL*, *LA*, and *LA*_*ref*_ are respectively the angles for Fixed Link, Lever Arm, and reference for Lever Arm computed from the reference disturbance torque.

The HAF module is schematically introduced in the control architecture, depicted in Figure 2. The controller is described by Equation (1).

(1) $$\begin{array}{r} u=\tau_{PID}+u_{HAF} \end{array}$$

where *u* is the output of the controller (pulse width modulation), τ_*PID*_ corresponds to the output of the torque controller (Equation 2), and *u*_*HAF*_ to the output of the haptic adaptive feedback module (Equation 3).

(2) $$\begin{array}{r} \tau_{PID}=K_{p}\cdot error+K_{i}\cdot \int_{0}^{t}error\cdot dt+K_{d}\cdot \frac{d}{dt}error \end{array}$$

(3) $$\begin{array}{r} u_{HAF}=K_{HAFi}\cdot \int_{0}^{t}\alpha \cdot dt+K_{HAFp}\cdot \alpha \end{array}$$

where *K*_*p*_, *K*_*i*_, and *K*_*d*_ are respectively the PID constants; *K*_*HAFi*_ and *K*_*HAFp*_ are respectively the integral and proportional constants of the HAF module; α angle is proportional to the interaction torque between human and robot; and *error* = *LA*_*ref*_ − *LA*. *LA* is the actual sensor information for the Lever Arm angle, whereas *LA*_*ref*_ is the calculated reference LA angle. This reference LA angle is calculated with the approximation of the MACCEPA actuator to a torsion spring actuator described by Equation (4).

(4) $$\begin{array}{r} LA_{ref}=\frac{\tau_{ref}}{K_{ts}}+FL \end{array}$$

where τ_*ref*_ is the reference disturbance torque to the controller, *K*_*ts*_ is the empirically obtained torsional stiffness constant, and *FL* is the Fixed Link angle, i.e., the user's ankle angle.

The objective of the controller is to apply higher disturbance torques when higher performance is reached (consequently adding more difficulty to the task), and vice versa (rendering the task easier with lower performances). To do this, *K*_*HAFi*_ and *K*_*HAFp*_ are empirically set to $\frac{K_{HAF}}{1000}$ and $\frac{K_{HAF}}{5}$, where *K*_*HAF*_ provides the modulation of the disturbance torque following this simple rule: *K*_*HAF*_ = 100 − performance [%], thus, the value of the constant *K*_*HAF*_ is updated based on task performance. Section 2.5.1 explains how this constant is modulated.

### 2.4. Protocol

**Figure 3:**
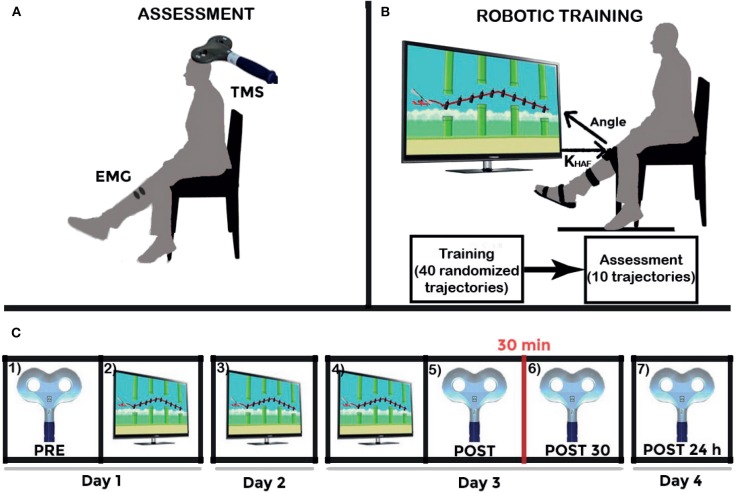
Experiment schematics. **(A)** Upper left figure shows the Transcranial Magnetic Stimulation (TMS) assessment setup. **(B)** Upper right figure shows the experimental setup together with the daily training structure: 40 training repetitions, and ten last repetitions to survey the execution after the training (at a settled disturbance torque, the maximum given by the robot: 15 N·m). And **(C)** Lower figure shows the longitudinal intervention structure: (1) TMS assessment (represented by the figure-of-8 coil) PRE-intervention; (2) first day training (represented by the visual paradigm); (3) second day training; (4) third day training; (5) TMS assessment POST-intervention; (6) POST30: TMS assessment 30 min after intervention; and (7) POST24h: TMS assessment 24 h after intervention. Adapted from Asín-Prieto et al. (2018), copyright 2019, Springer Nature Switzerland AG.

The longitudinal intervention protocol applied on each participant is graphically described in Figure 3. The intervention lasted 4 days. Training sessions were performed in days 1–3. Four corticospinal assessments were performed in days 1, 3 (two assessments), and 4.

**Figure 4:**
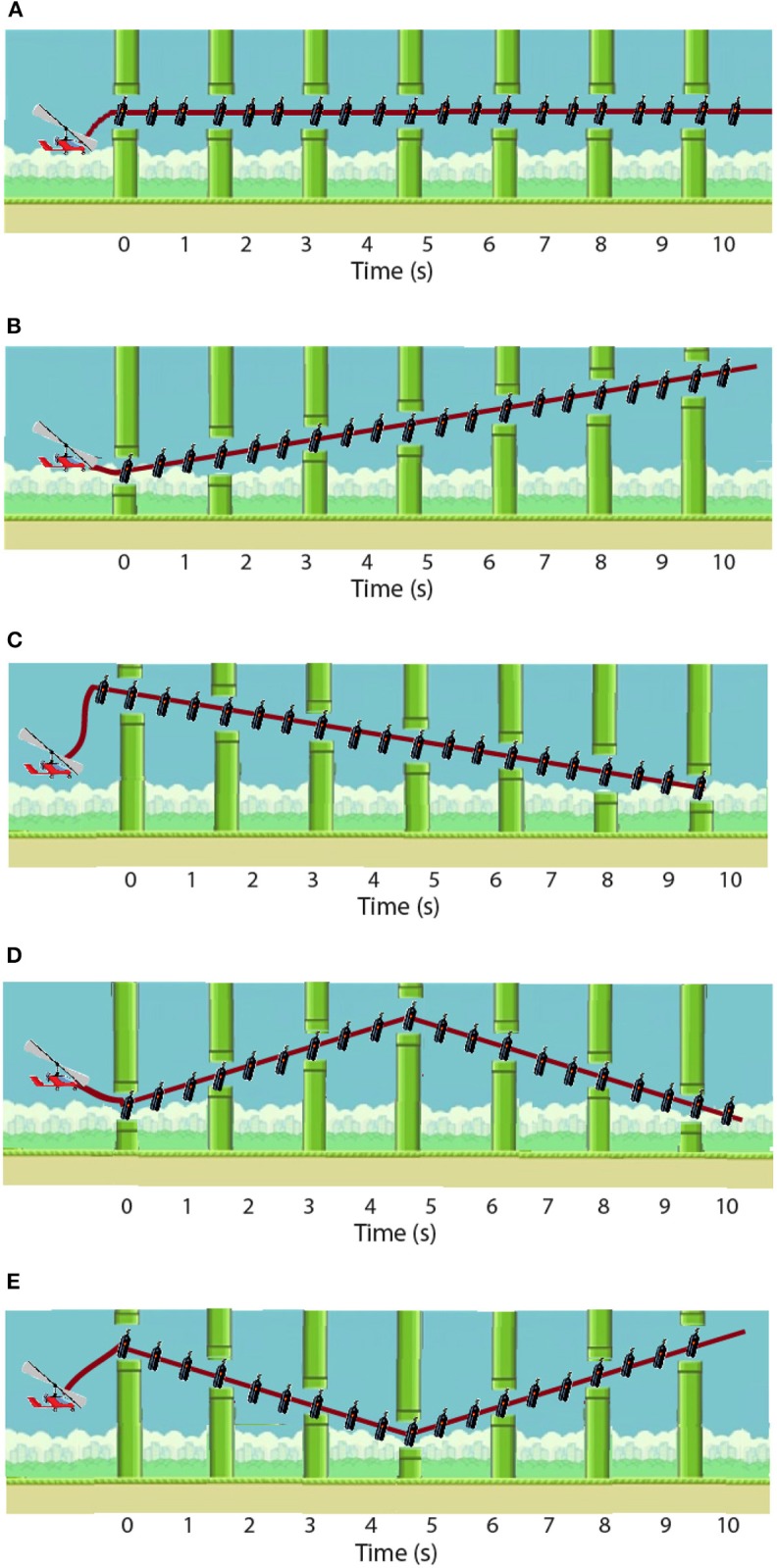
Five possible trajectory profiles: **(A)** constant −2.5°; **(B)** straight increasing from −4 to 1°; **(C)** straight decreasing from −1 to −6°; **(D)** from −6 to −2.5 and back to −6 again; and **(E)** from 1 to −2.5 and back to 1. Modified from Asín-Prieto et al. (2019), copyright 2019, IEEE.

**Figure 5:**
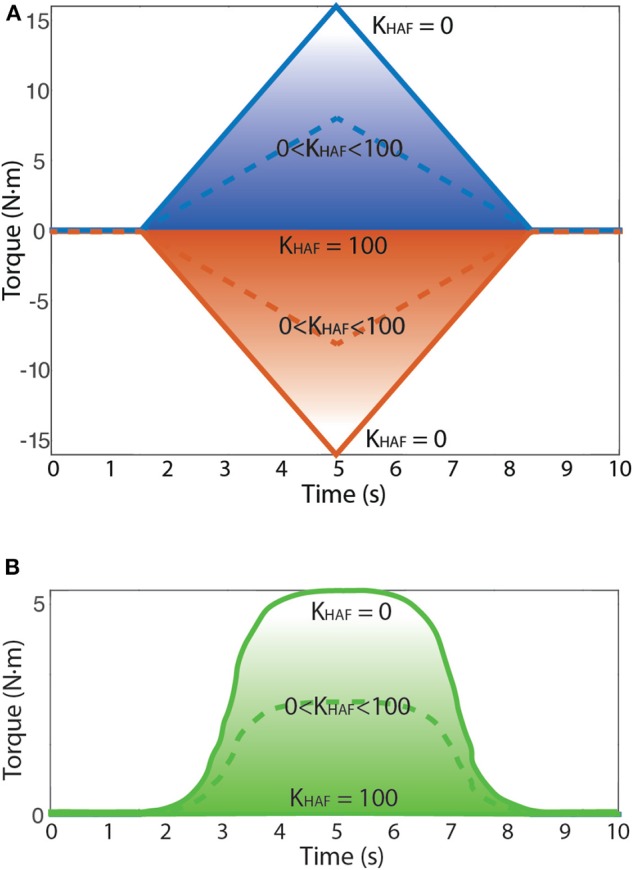
The behavior of the HAF module is depicted: (1) *K*_*HAF*_ = 100 prompts zero-torque control; (2) *K*_*HAF*_ = 0 normal torque control, no influence of HAF, so up to 15 N·m reference; and (3) *K*_*HAF*_ between 0 and 100, nearer to a zero-torque control the higher the constant *K*_*HAF*_ is, thus allowing to modulate the magnitude of the applied disturbance torque amplitude. Dashed line corresponds to an example of disturbance torque profile with *K*_*HAF*_ between 0 and 100. **(A)** Possible disturbance torque profiles: torque to dorsiflexion (up) and torque to plantarflexion (down). **(B)** Possible disturbance torque to dorsiflexion (up) direction for the patient.

The training follows this daily structure: forty training repetitions (randomized trajectory profiles, as shown in Figure 4), disturbance torque modulated by the system; followed by ten evaluation repetitions [two types of disturbance torque profiles—Figure 5A, multiplied by the five possible trajectories—Figure 4] for the assessment of immediate effect. The disturbance torque provided in these assessment repetitions was set at the maximum given by the robot: 15 N·m. All repetitions had a duration of 10 s per trajectory. The resting position of the ankle was set at −2.5° (slightly plantarflexed) as the most comfortable position for the users.

The task instruction was to follow the trajectories delineated in the visual paradigm by means of the sequence of onscreen items (gas bottles) following the shortest linear path in-between. The user had to move a character (gyrocopter) with the angular position of the ankle via dorsi-plantarflexion to collect the gas bottles: dorsiflexion implied moving the avatar upwards in the screen, whereas plantarflexion implied going downwards. Meanwhile, the robot disturbed the user motion by performing plantar and dorsiflexion alternated disturbance torque profiles (see Figure 5A). These disturbance torque profiles were developed with the aim of stimulating both agonist and antagonist muscle groups, both in dorsi- and plantarflexion movements.

For the patient, we focused only on dorsiflexion disturbance torque patterns, because he was unable to avoid the full drop of the foot. Besides, the disturbance torque was modified (as seen in Figure 5B) to remove abrupt changes in the direction of the force exerted by the robot. We empirically set a maximum disturbance torque of 5 N·m.

All healthy subjects were asked to train and find a strategy to actively compensate the disturbance torque by the ankle robot, to successfully follow the trajectory on the screen, along three sessions (one every day), of 50 repetitions. For the patient, the length of the protocol was modified to 5 days (replicating the protocol used in Asín-Prieto et al., 2018).

### 2.5. Metrics

#### 2.5.1. Robot-Based

**Figure 6:**
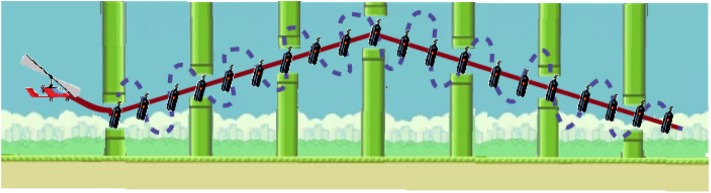
Trajectory example, with the best trajectory between items in continuous red line, and a high error trajectory between items (with 100% SCORE as all the items are collected) in dotted blue line.

**Figure 7:**
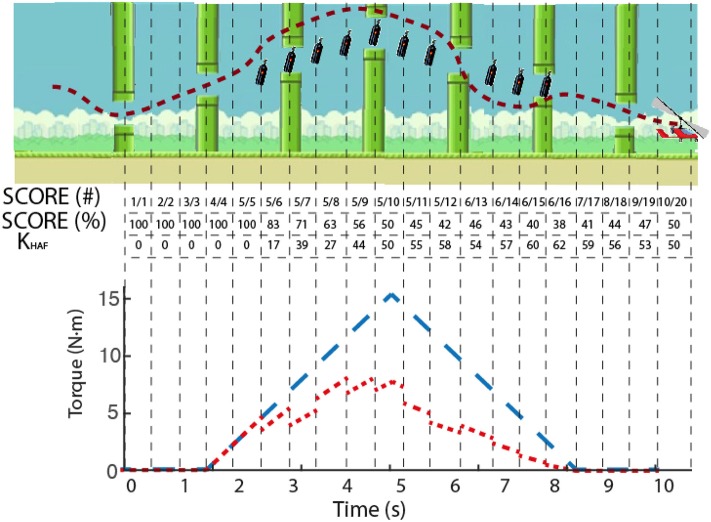
Example of the modulation of *K*_*HAF*_. Dashed line in the upper panel depicts the actual trajectory followed by a subject, with the uncollected bottles remaining onscreen. The instantaneous SCORE is presented in the table, both in collected/total (#) and percentage (%), as well as the computed value for *K*_*HAF*_. Lower panel shows in blue the reference torque (corresponding to a SCORE of 100%), and in red the actual reference disturbance torque applied to the user's ankle modulated according to the SCORE.

We used two different metrics to quantify the performance of the user: SCORE and root mean squared error (RMSE). SCORE was calculated for each trial as the percentage of collected onscreen items, whereas RMSE was calculated by subtracting the performed trajectory from an ideal linear path between onscreen items. Note that it could be possible to collect all the onscreen items by performing a high error trajectory between them (see Figure 6 for an example). The total SCORE for each trial was shown to encourage the user to improve it along the session. The value for *K*_*HAF*_ was updated when the gyrocopter exceeded a (collected or uncollected) gas bottle, based on the instantaneous SCORE in the current trial, thus modulating the disturbance torque. Each trial consisted of 20 collectible bottles, thus rendering a *K*_*HAF*_ refresh rate of 2 Hz (20 gas bottles per 10 s). Figure 7 depicts an example of the modulation of the disturbance torque based on the SCORE metric.

**Figure 8:**
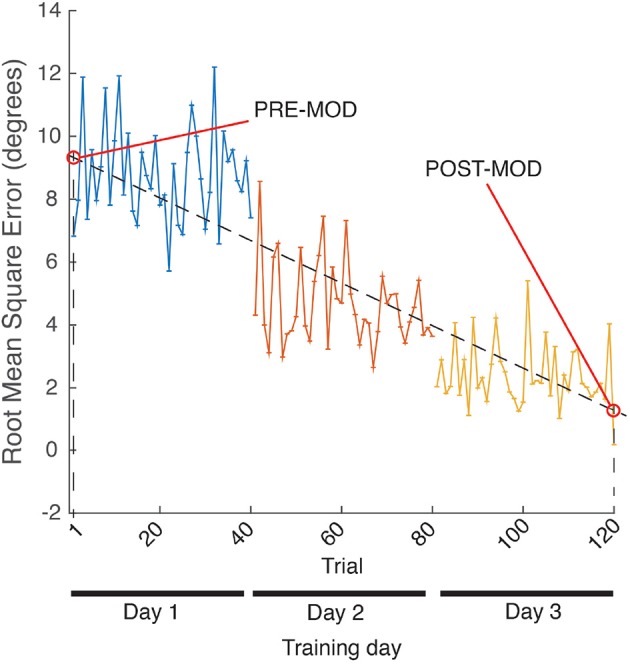
MOD metric calculation example, for RMSE.

SCORE and RMSE were used to quantify two different sets of data: (a) assessment post-training repetitions, i.e., the 10 last repetitions of each training day (see ROBOTIC TRAINING in Figure 3), in what we called POST-train values; (b) linear fit on the sequence of the 120 training repetitions (40 training repetitions per day, concatenated for the 3 days), and selected the values of the resulting linear fitting coinciding with the first (1) and last (120) repetitions, in what we called MOD (modulated) values, where PRE-MOD and POST-MOD were the first and last values of the linear fit, respectively (see Figure 8).

In addition to RMSE and SCORE after each day training (POST-train), we used two other metrics for the patient: changes in range of motion (ROM) and velocity. Before and after the training from the second to the fifth day, the patient underwent a robotic evaluation of the possible ROM. This evaluation consisted on moving up and down a ball on the screen via dorsi-plantarflexion during 30 s. The patient was asked to alternatively reach two horizontal lines (one up and one down), and the position of these lines was changed to the maximum reached in order to make the task more difficult. Although the separation between lines meant a wider ROM, the absolute position of them remained the same onscreen in order to be unnoticeable for the patient. The maximum velocity was calculated by multiplying the maximum achieved angular amplitude by the fundamental frequency (calculated with Fast Fourier Transform). We computed the change in this metric by comparing the results before and after the intervention.

#### 2.5.2. Clinical Assessment

In the rehabilitation process there are three main phases that need to be characterized: (1) initial assessment, to identify and measure the extent of the pathology; (2) planning, to assess the problem and establish the objectives; and (3) final assessment, after the treatment. In addition to the aforementioned metrics, the clinicians at *CEADAC* performed a functional clinical assessment at the beginning and the end of the week for the patient, before the first training session, and after the last one. In the functional assessment protocol developed in *CEADAC*, among the broad set of clinical functional scales that aim to provide an objective insight in the recovery process of patients, the clinicians focus on: Timed 10 m walk, as a measure of gait speed; 6 Min Walking Test (6MWT), as a measure of resistance; Step Test, as a measure of dynamic balance; Timed Up and Go (TUG) test, that demands several potentially destabilizing maneuvers for the subject.

#### 2.5.3. Neurophysiological Assessment

Corticospinal excitability was assessed by recording the MEPs elicited by a Magstim 200^2^ TMS stimulator in single pulse modality in combination with a figure-of-eight double-coned coil. We followed the instructions by SENIAM (Hermens et al., 1999) to place the surface electromyography Ag/AgCl electrodes (22.225 × 34.925 mm, Vermed), recorded with a g.USBamp amplifier (g.tec), sampled at 24 KHz and highpass filtered with a 20 Hz first order Butterworth filter.

In order to map the hot spot [place where Tibialis Anterior (TAnt) MEPs peak-to-peak amplitude is higher] on the scalp, several supra-threshold pulses were delivered nearby the vertex. The hot spot, ineon, and vertex were drawn with a permanent marker on a swimming cap, in order to ensure repeatability between sessions. After locating the hot spot, the resting motor threshold (RMT), defined as the stimulation intensity that elicits MEPs of ~50 μV peak-to-peak amplitude in 5 out of 10 applied pulses (Temesi et al., 2014), was set for each participant. We recorded ipsilaterally (Kamibayashi et al., 2009) TAnt and Soleus (SO), as well as Rectus Femoris (RF, as a control muscle not involved in the robotic ankle task).

The assessment consisted in delivering 20 pulses to each of the volunteers at an intensity of 120 % of the RMT to elicit MEPs. The peak-to-peak amplitude of the MEPs was averaged. This assessment procedure was performed four times (see Figure 3): (1) before the training of the first day (PRE); immediately after the training of the third day (last training, POST); 30 min after to evaluate plastic effects (POST30); and finally 24 h after, in order to check lasting effects (POST24h).

#### 2.5.4. Satisfaction Questionnaire

After the treatment, all subjects filled out a Likert scale (1–Very unsatisfied; 2–Unsatisfied; 3–Not satisfied nor unsatisfied; 4–Satisfied; 5–Very satisfied) questionnaire for assessing the satisfaction level with the experimental procedure.

### 2.6. Data Analysis

Data were analyzed with Matlab, IBM SPSS Statistics version 25, and R Studio. After examining with Shapiro-Wilk test, our data showed variables with normal distributions and variables violating the normality. Thus, for those without a normal distribution, we provide the results for non-parametric tests; and for those that present a normal distribution, we provide parametric analyses.

First of all, changes in RMSE POST-train metric, both for healthy subjects and the patient, were tested using a Friedman test of differences among repeated measures along the study, finally evaluating the size effect with Average Spearman rho ($\bar{\rho_{s}}$), and performing a Pairwise *post-hoc* Test for Multiple Comparisons of Rank Sums for Unreplicated Blocked Data (Conover-test) with Bonferroni correction. For the SCORE POST-train, both for healthy subjects and the patient, we performed a One-way repeated measures ANOVA, with Huynh-Feldt correction due to lack of sphericity (Mauchly's test), with partial squared omega ($\omega_{p}^{2}$) for the size effect, and pairwise *t*-test *post-hoc* analysis, with Bonferroni correction.

Then, we tested the correlation between SCORE and RMSE with a Spearman bivariate analysis (p value of 0.05), for the evaluation ratings after each day training (POST-train), to check the relationship between metrics.

To assess changes in the PRE-MOD vs. POST-MOD of the SCORE and RMSE on the modulated repetitions, we conducted *t*-Student analyses, providing Cohen's *d* as the size effect.

Finally, changes in the corticospinal excitability were also tested using a Friedman test, and evaluating the size effect with Average Spearman rho, and performing Conover *post-hoc* Test with Bonferroni correction.

## 3. Results

Data showed normal distribution for: SCORE POST-train both for healthy subjects and the patient, and MOD for SCORE and RMSE. All the other variables presented a non-normal distribution (see Table 1 for the descriptive statistics).

**Table 1:** Descriptive statistics for the variables analyzed for the group of healthy individuals.

		**Mean**	**Median**	**Standard deviation**	**Min**	**Max**
RMSE POST-train	1st day	3.22	2.82	2.31	1.52	9.59
	2nd day	2.43	2.39	1.05	1.23	4.95
	3rd day	2.24	2.14	0.99	1.11	4.57
SCORE POST-train	1st day	54.65	54.25	19.53	15.50	84.50
	2nd day	65.15	62.25	9.92	55.00	82.50
	3rd day	68.00	64.75	11.97	54.00	87.00
RMSE modulated	PRE-MOD	4.02	3.65	1.77	1.62	6.86
	POST-MOD	1.99	1.79	0.95	0.98	4.05
SCORE modulated	PRE-MOD	43.54	40.15	18.34	24.07	80.11
	POST-MOD	67.84	66.77	9.77	54.92	85.05
TAnt MEPs	PRE	238.99	176.33	144.07	107.16	505.69
	POST	312.94	344.67	154.82	108.96	504.29
	POST30	281.59	296.33	111.02	107.32	411.23
	POST24h	403.56	385.64	222.82	114.94	807.08
SO MEPs	PRE	94.36	90.51	43.42	28.33	162.03
	POST	109.00	119.30	50.21	30.48	169.56
	POST30	99.35	100.20	44.19	29.41	176.05
	POST24h	107.46	115.84	50.21	31.87	198.76
RF MEPs	PRE	228.17	209.44	163.19	34.49	495.57
	POST	215.42	243.36	129.98	34.38	387.78
	POST30	190.06	146.14	171.15	22.50	555.03
	POST24h	236.82	210.45	164.92	31.45	487.19
Satisfaction	4.80	5.00	0.42	4.00	5.00	

### 3.1. Study With Healthy Individuals

**Figure 9:**
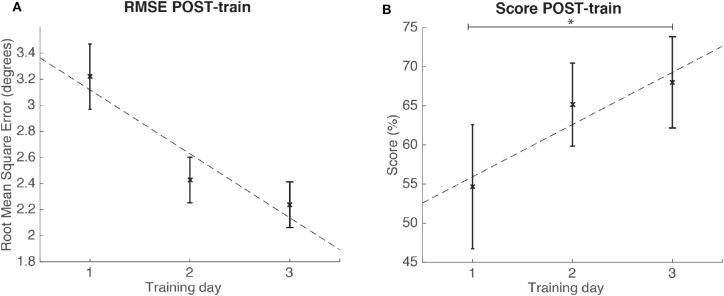
Results for the RMSE and SCORE after each day training (POST-train) for the group of healthy individuals. Statistical significance (*post-hoc* comparison) depicted by the asterisk (*). **(A)** Mean and standard error of POST-train for RMSE per day. **(B)** Mean and standard error of POST-train for SCORE per day.

There was a significant change in SCORE POST-train metric [ANOVA, F_(1.18, 10.60)_ = 6.84; *p* < 0.05; $\omega_{p}^{2}$ = 0.35; large effect size according to Field, 2018] but not in RMSE POST-train (*p* > 0.05). *Post-hoc* tests revealed that the SCORE at the third training day was significantly increased (*p* = 0.03) as compared to the SCORE on the first training day (see Figure 9).

We found significant (*p* < 0.05) strong correlations (ρ > 0.70) in the evaluation ratings after each day training (POST-train); both for SCORE 1st day and RMSE 1st day (ρ = −0.89), and SCORE 3rd day and RMSE 3rd day (ρ = −0.86).

*t*-Student indicated that the SCORE POST-MOD of the modulated training was significantly higher than the SCORE PRE-MOD [*t*(9) = −4.39; *p* < 0.05; Cohen's *d* = 1.39], and that the RMSE POST-MOD was significantly lower than the RMSE PRE-MOD [*t*(9) = 3.05; *p* < 0.05; Cohen's *d* = 0.96]. There was a large size effect for both metrics' *t*-tests according to Kotrlik and Williams (2003).

**Figure 10:**
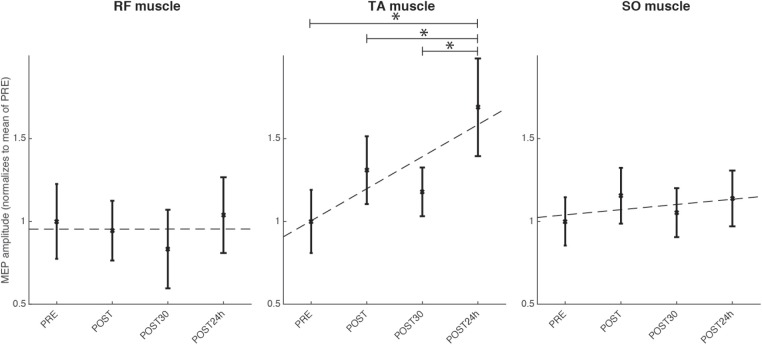
Results for the TMS assessment for the group of healthy individuals for the four evaluated moments, normalized to the mean of the evaluation before the intervention: before the full intervention (PRE), right after the full intervention (POST), 30 min after the POST (POST30), and 24 h after the end of the full intervention (POST24h); for the muscles TAnt (Tibialis Anterior), RF (Rectus Femoris) and SO (Soleus). Statistical significance (*post-hoc* comparison) depicted by the asterisk (*).

TAnt MEPs peak-to-peak amplitude was significantly changed [Friedman, χ^2^ = 9.12; *p* < 0.05; 3 DoF; $\bar{\rho_{s}}$ = 0.22; small effect size according to Kotrlik and Williams (2003)] across assessment sessions. *Post-hoc* tests revealed that TAnt MEPs peak-to-peak amplitude was significantly increased at the POST24h moment when compared to PRE (*p* < 0.01), POST (*p* = 0.03), and POST30 (*p* < 0.01) moments (see Figure 10). On the other hand, there was no significant change in SO nor in RF MEPs peak-to-peak amplitude across assessment moments (*p* > 0.05).

The satisfaction questionnaire rendered an average of 4.8 (being 5 Very satisfied), with a standard deviation of 0.42.

### 3.2. Usability Case Study With Post-stroke Patient

**Figure 11:**
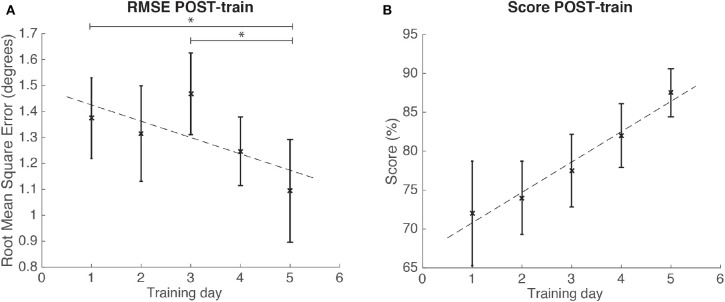
Results for the RMSE and SCORE POST-train, after each day training for the patient. Statistical significance (*post-hoc* comparison) depicted by the asterisk (*). **(A)** Mean and standard error of POST-train for RMSE per day. **(B)** Mean and standard error of POST-train for SCORE per day.

In the case study, we used the repetitions for each of the 5 training days to conduct the statistical analyses, as we had data from one individual. The results for the case study rendered significant changes in RMSE POST-train [Friedman, χ^2^ = 9.36; *p* = 0.05; 4 DoF; $\bar{\rho_{s}}$ = 0.14; medium effect size according to Kotrlik and Williams (2003)]. *Post-hoc* tests revealed that RMSE at training day 5 was significantly decreased when compared to day 1 (*p* < 0.01) and day 3 (*p* = 0.02) (see Figure 11). On the other hand, the SCORE did not show significant differences (ANOVA, *p* = 0.09) across training days (see Table 2 for the descriptive statistics).

**Table 2:** Descriptive statistics for the RMSE (°) and SCORE (%) POST-train for the patient.

		**Mean**	**Median**	**Standard deviation**	**Min**	**Max**
RMSE POST-train	1st day	1.37	1.21	0.49	0.65	2.45
	2nd day	1.32	1.29	0.58	0.12	2.36
	3rd day	1.47	1.19	0.50	1.05	2.43
	4th day	1.25	1.06	0.42	0.97	2.31
	5th day	1.09	1.00	0.63	0.31	2.22
SCORE POST-train	1st day	72.00	72.50	21.24	35.00	100.00
	2nd day	74.00	75.00	14.87	50.00	100.00
	3rd day	77.50	77.50	14.77	55.00	100.00
	4th day	82.00	85.00	12.95	55.00	100.00
	5th day	87.50	90.00	9.79	75.00	100.00

**Figure 12:**
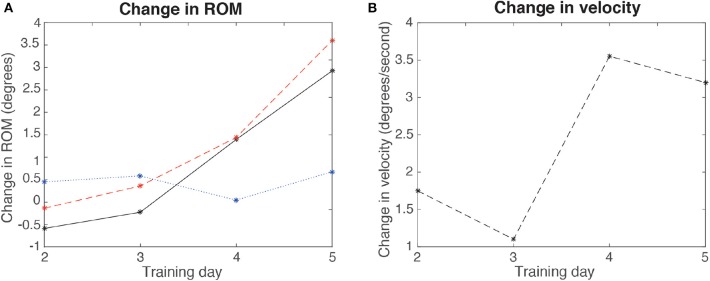
Results for the change in ROM and velocity per day (comparing before and after each day training), for the second, third, fourth, and fifth days, for the patient. **(A)** ROM (continuous line), maximum angles (dashed line), and minimum angles (dotted line) difference (POST-PRE) per day. **(B)** Velocity change per day.

Figure 12 shows the results of the evaluation of ROM and velocity before and after each training session (from the second to the fifth day). There was a decreased ROM in days 2 and 3, and increased ROM in days 4 and 5, with a positive trend across days. A different trend was obtained in velocity (there was a decrease between the second and the third day, and between the forth and the fifth, although there was a net increase in velocity). The figure also shows the difference of the maximum and minimum values of achieved ROM per day, showing that the maximum increased across days.

Table 3 presents the improvements on the functional scales, before and after the full treatment. Modified Ashworth scale was used to assess the level of spasticity of the patient, showing no changes in muscle tone.

**Table 3:** Clinical scales before the beginning and after the end of the full 5 days treatment.

	**Before**	**After**
Timed 10 m walk (m/s)	1.6	1.7
6MWT (m)	455	465
Step test (repetitions)	7	11
TUG (s)	9.50	8.52

The satisfaction questionnaire for the patient showed that he was very satisfied with the intervention.

## 4. Discussion

We aimed at exploring the validity of combining the robotic ankle exoskeleton with a video game designed to promote motor learning in a therapy protocol involving autonomously customized control. We have developed the video game to enhance adherence and engagement, by providing the control of the robot with the performance to modulate the task difficulty. We approached this objective by providing perturbations to the users' ankle while asking them to follow a trajectory depicted as a sequence of collectible onscreen items. The magnitude of the perturbations was modulated as function of the performance, i.e., the number of collected items, making the task more difficult if the performance increased, and vice versa.

This reward system, based on autonomously customized hardness of the task to the user, potentially promotes learning. We also computed the error as the difference between the most efficient trajectory between onscreen items and the actual performed trajectory. Moreover, we evaluated other metrics (MEPs for the corticospinal excitability for healthy individuals; clinical scales, range of motion and velocity for the patient; and satisfaction with the process of intervention for all participants) to support our novel approach to a clinical therapy.

Regarding TMS, although there are limitations and results are not consistent when extrapolating corticospinal excitability improvement to learning processes in rehabilitation (Carson et al., 2016), several recent studies point out that an increase in corticospinal excitability may be related to an improvement in motor learning (Kida et al., 2016; Naros et al., 2016; Mawase et al., 2017; Christiansen et al., 2018; Raffin and Siebner, 2018; Mrachacz-Kersting et al., 2019), and moreover, there is a relationship between the improvement in the metrics in the robotic therapy, motor learning, and corticospinal excitability enhancement in healthy subjects (Perez et al., 2004). For this reason, we decided to use TMS as a valid technique to assess the corticospinal excitability.

Regarding the validation study in healthy volunteers, we found significant improvement in the SCORE for the POST-train metrics, but not for the RMSE, although there was a strong significant negative correlation between SCORE and RMSE. would imply that similar results These results are in line to those already found by us with a single volunteer (Asín-Prieto et al., 2018), and suggested that participants would learn and master the robotic task across days.

We tested MOD metric as a customized modality for the assessment of the performance, as the controller (and thus the disturbance torque applied to the ankle) was autonomously modulated via HAF algorithm, rather than applying the maximum disturbance torque (15 N·m), as it was done for the POST-train metric. Thus, we considered the MOD metric as a more appropriate way to assess the personalized performance. In contrast to the POST-train metrics, we found significant improvements along the training in the MOD variable for both the SCORE and RMSE.

We also found a significant increase in TAnt MEPs peak-to-peak amplitude, supporting the hypothesis that an increase in performance has a relationship with corticospinal excitability. These corticospinal changes did not show muscle specificity, as our training involved only the ankle and we have not found statistical changes in RF (control muscle) neither in SO corticospinal excitability. Consequently, we can only conclude that our training lead to increased TAnt excitability. We can speculate that one of the reasons why SO had not significantly increased corticospinal excitability, may be the fact that the robot controller is in favor of gravity, and thus the force to move the robot downwards requires less muscle activation than the required TAnt activation to dorsiflex the ankle. Another possible explanation could be that TAnt, according to Brouwer and Ashby's findings (Brouwer and Ashby, 1992), presents higher corticospinal projections density than the rest of the lower-limb muscles, and thus it may be easier to assess its excitability. In this sense, corticospinal projections to TAnt in comparison to the rest of lower-limb distal muscles, are comparable to those at upper-limb level (Brouwer and Ashby, 1990), and thus we could say that our results are consistent to those in the literature for upper-limb robotic approaches (Ramos-Murguialday et al., 2014; Kraus et al., 2016). Finally, another reason for different changes in corticospinal excitability of TAnt and SO may be that TMS can possibly activate inhibitory projections (that present a lower threshold than excitatory projections) (Nielsen and Kagamihara, 1993) that are richer in plantarflexor muscles (SO) (Hudson et al., 2013). Therefore, even if corticospinal enhancement occurs for SO, this would probably not be easily observed (Fujio et al., 2019).

In the case study (with the post-stroke patient) results, we found significant improvements in the RMSE after each day training, but non-significant changes for the SCORE. The significant changes found on RMSE are consistent not only with the results of our healthy sample, but also with the results presented by others (Patton et al., 2001; Krakauer, 2006; Reinkensmeyer and Patton, 2009; Goodman et al., 2014), thus confirming our hypothesis of usability in the case study. Although we have provided some data on the performance for this patient, our main goal was to validate the usability of this robotic training framework for post-stroke rehabilitation.

When we compared the ROM and velocity before and after each day training, we found that there was a net increase both in velocity and ROM along the days, although in the velocity there was a sawtooth shape profile (third and fifth day presented a lower increase than second and fourth, respectively). Nonetheless, as higher ROMs would inevitably render lower velocities (as it depends on the maximum achieved angular amplitude and the fundamental frequency of the resulting signal, and thus a higher amplitude would decrease the speed and vice versa), the sawtooth-shaped behavior of the velocity could be explained by this phenomenon. Furthermore, the fact that the maximum dorsiflexion increased across days may indicate an improvement in dorsiflexor muscles. If these data imply a behavioral improvement in the control of the ankle, the variation occurs together with that of the clinical scales used to assess the improvement after the treatment. Consequently, we can consider that these changes in ROM and velocity tend to improve like the clinical scales.

As reported by the Likert satisfaction scale, both studies lead to full satisfaction of the participants. We found the viability of using this treatment in patients, as the patient ranged the intervention similarly to the range given by the healthy group.

Our objective was to validate our proposed therapy as a potential tool for increasing motor learning on healthy individuals. Thus, taking into consideration all these results, our hypothesis has been confirmed in the POST-train metric for the SCORE and for the TAnt excitability; and for the MOD variables. Regarding RMSE, our hypothesis has not been confirmed, probably due to the short duration of the treatment. On the other hand, for the case study with the post-stroke patient, both SCORE and RMSE have changed as hypothesized in the design of the study.

Finally, we conclude that combining a grounded exoskeleton that disturbs the ankle joint motion with a video game incorporating autonomously controlled difficulty can elicit improvements on performance, and also increased excitability of the target muscle(s). This conclusion renders our proposal as a potential rehabilitation tool. Furthermore, we have demonstrated the viability of applying this treatment approach in a usability case study with a post-stroke patient.

As future work, we aim at extending this study using more stroke patients. This rehabilitation approach may be also explored as a novel rehabilitation framework to be used in other pathologies like spinal-cord injury (Asín-Prieto et al., 2016), cerebral palsy (Lambrecht et al., 2014; Lefmann et al., 2017), or other lower limb movement disorders (Reinkensmeyer et al., 2004; Calabro et al., 2016).

### 4.1. Limitations

One limitation of the case study here presented is that we only enrolled a single stroke patient. Although improvements have been shown in task performance as observed in all assessed metrics, we cannot conclude that these improvements are only due to our treatment, as the patient was also enrolled on his daily therapy with physio and occupational therapists. Nonetheless, both clinical and robot-based metrics rendered a good prospective of the integrated therapy, which should be explored by us in a wider population of stroke patients, for longer therapy sessions.

To avoid very long daily sessions for the healthy controls, we discarded the option of assessing corticospinal excitability before and after each robotic-training session. Thus, we cannot isolate the daily effects of the robotic training.

Furthermore, due to the tight therapy schedule of the patient, and to avoid lengthy sessions, the professionals at CEADAC decided to remove the TMS assessment from the protocol.

Results obtained from TMS assessments partially demonstrate the effectiveness of the robotic-therapy in modulating the corticospinal excitability in the healthy group, since the MEPs were significantly increased only in TAnt but not in SOL muscle. Although TMS presents some limitations as a diagnostic tool, namely the inter-subject variability in simultaneous measurements on normal population (Choudhury et al., 2011), the intra-subject variability obtained in our study was relatively small. On the other hand, it is worth noting that our work adds more evidence to other studies showing that different tasks may lead to increased TA excitability but not to increased SOL or Gastrocnemius Medialis (MG) excitability. For instance, results presented by Fujio et al. (2019) suggest that TA excitability is susceptible to the prediction of a perturbation, whereas the SO and MG excitability presented no change for the same tasks. In any case, we plan to assess data from a control group that does not perform the robotic treatment done by the healthy subjects in this study.

We are also planning to improve the neurophysiological assessment by including other technologies, such as assessment of changes in spinal reflexes (e.g., reciprocal inhibition) (Pascual-Valdunciel et al., 2019) or paired-pulses TMS protocols. By combining these technologies, we should be able to have a better understanding on the level (spinal, supraspinal, or both) where of plastic changes occur due to the robotic therapy.

## Data Availability Statement

The datasets generated for this study are available on request to the corresponding author.

## Ethics Statement

The studies involving human participants were reviewed and approved by Bioethical Subcommittee of the Ethical Committee of CSIC (Spanish National Research Council). The patients/participants provided their written informed consent to participate in this study.

## Author Contributions

GA-P, AM-E, FB, EU, JP, and JM: main writing process for the manuscript. GA-P, AM-E, FB, JM, JG-V, SS, and FA: contribution to study conceptualization. GA-P, AM-E, FB, and JM: study design. GA-P, AM-E, and CG-A: data acquisition. GA-P and AM-E: data analysis. GA-P, AM-E, FB, and JM: data interpretation. All authors: approval of final manuscript and agreement to be accountable for all aspects of the work while ensuring that questions related to the accuracy or integrity of any part of the work are appropriately investigated and resolved.

### Conflict of Interest

The authors declare that the research was conducted in the absence of any commercial or financial relationships that could be construed as a potential conflict of interest.
